# Optimization of PID trajectory tracking controller for a 3-DOF robotic manipulator using enhanced Artificial Bee Colony algorithm

**DOI:** 10.1038/s41598-023-37895-3

**Published:** 2023-07-10

**Authors:** Muhammad I. Azeez, A. M. M. Abdelhaleem, S. Elnaggar, Kamal A. F. Moustafa, Khaled R. Atia

**Affiliations:** 1 Mechanical Design and Production Engineering Department, Zagazig University, Zagazig, 44519 Egypt; 2 Industrial Engineering Department, Zagazig University, Zagazig, 44519 Egypt

**Keywords:** Mechanical engineering, Mathematics and computing

## Abstract

This study introduces and compares two optimization techniques, the basic Artificial Bee Colony (ABC) and the enhanced Artificial Bee Colony with multi-elite guidance (MGABC), for determining optimal gains of a Proportional-Integral-Derivative (PID) controller in a 3 degrees of freedom (DOF) rigid link manipulator (RLM) system. The objective function used in the optimization process is a novel function that is based on the well-known Lyapunov stability functions. This function is evaluated against established error-based objective functions commonly used in control systems. The convergence curves of the optimization process demonstrate that the MGABC algorithm outperforms the basic ABC algorithm by effectively exploring the search space and avoiding local optima. The evaluation of the controller's performance in trajectory tracking reveals the superiority of the Lyapunov-based objective function (LBF), with significant improvements over other objective functions such as IAE, ISE, ITAE, MAE and MRSE. The optimized system demonstrates robustness to diverse disturbance conditions and uncertainty in the mass of the payload, while also exhibiting adaptability to joints flexibility without inducing any vibrations in the movement of the end-effector. The proposed techniques and objective function offer promising avenues for the optimization of PID controllers in various robotic applications.

## Introduction

Approximately seven decades ago, the development of robotic manipulators emerged as a viable solution to replace human workers in hazardous industrial settings. These robots are commonly employed in inaccessible areas where repetitive tasks need to be executed within specific timeframes. One of the essential applications for mechanical robotic manipulators is picking and positioning material^1^. Furthermore, industrial manipulators find utility in handling radioactive and biohazardous materials during robot-assisted surgical procedures, as well as performing various functions such as welding, assembly, manufacturing, painting, and other operations within the automotive industry^2–4^.

Mathematical modeling plays a crucial role in understanding and optimizing the behavior of multi-degree-of-freedom (MDOF) robotic manipulators in industrial applications. The development of a mathematical model for such mechanisms often employs the Lagrangian approach^5–8^. However, the process of mathematical modeling poses significant challenges, particularly for nonlinear systems, and involves intricate and time-consuming calculations. Lee and Alandoli^3^ have conducted a comprehensive analysis of various mathematical modeling techniques, offering valuable insights into this field.

The application of SimMechanics models in robot modeling using Simulink and Simscape toolboxes, employing numerical modeling techniques, has been widely adopted in research^2, 9, 10^. These models offer advantages such as simplicity and controllability. Simulation software enables researchers to gain a deeper understanding of the behavior of Multi-Degree-of-Freedom (MDOF) robotic manipulators in a simulated environment. Furthermore, utilizing simulation software helps mitigate the complexities associated with mathematical formulations. However, upon conducting a literature review, it becomes evident that there is a scarcity of research focused on this specific area, particularly in terms of validating mathematical models with Simscape^11, 12^. Additionally, there is a lack of validation for models created using other software tools like MSC Adams. These findings highlight the importance of further attention and investigation in this field.

By addressing this research gap, the current study contributes to the existing body of knowledge by providing validation for the mathematical models using both Simscape and MSC Adams. This validation process enhances the credibility and reliability of our research findings. Furthermore, it serves as a steppingstone for future research endeavors in the field of robotic manipulator systems.

Controlling robot manipulators presents a highly intriguing domain due to the complex nature of their dynamical models. The dynamical analysis of robotic models involves examining the relationship between the arm's positions and the joint torques exerted by the actuators. Achieving precise and dependable control becomes challenging due to the interconnected relationships and nonlinear dynamics inherent in these systems. Consequently, the development of a controller using conventional control techniques that rely on the system's dynamics becomes a formidable undertaking^6, 13^.

The proportional-integral-derivative (PID) control system is extensively employed in diverse industrial domains owing to its simplicity and effectiveness^13–19^. Furthermore, the global asymptotic stability of linear PID controllers has been demonstrated for uncertain robotic manipulators through the utilization of Lyapunov's direct method and LaSalle's invariance principle^20, 21^. Moreover, the global asymptotic stability of linear PID controllers has been established for a point mass subjected to Coulomb friction by employing a discontinuous Lyapunov-like function and an appropriate application of LaSalle's invariance principle^22^.

The tuning of PID controller gains plays a crucial role in enhancing system performance and efficiency, as the tuning rule enables optimal disturbance rejection within the PID control feedback loop^23^. Traditional optimization techniques, such as Ziegler-Nichols approaches, often fail to yield satisfactory results when tuning PID controller gains. In recent years, evolutionary algorithms (EAs) have emerged as effective and efficient optimization techniques for addressing practical optimization problems encountered in scientific research and engineering applications. This is particularly relevant as many modern practical optimization problems exhibit non-convexity, discontinuity, and non-differentiability, posing challenges for conventional optimization techniques, such as gradient-based approaches^24–27^.

Swarm-based optimization algorithms (SOAs) utilize natural processes to guide the search towards the optimal solution. Unlike conventional algorithms like hill climbing and random walk, SOAs operate on a population of solutions rather than a single solution per iteration. This fundamental difference sets SOAs apart from these algorithms, as each iteration involves processing a population of solutions and generating a new population of solutions^28^. Various distinct paradigms are employed within evolutionary algorithms, including Jellyfish Search Optimization (JSO), Whale Optimizer Algorithm (WOA), Grey Wolf Optimizer (GWO), Ant Colony Optimization (ACO), Particle Swarm Optimization (PSO), Cuckoo Search Optimization (CSO), and Artificial Bee Colony (ABC) algorithm, among others. These paradigms showcase the diversity and effectiveness of evolutionary algorithms in solving optimization problems.

Elkhateeb and Badr^29^ employed the ABC (Artificial Bee Colony) optimization algorithm to determine the optimal gains of a PID controller for a 2DOF robotic manipulator. The tuning process involved the utilization of three distinct objective functions: the mean of the root of square error (MRSE), mean absolute error (MAE), and reference-based error with control effort (RBECE). However, the evaluation of the controller's robustness was limited to its performance in the presence of disturbance, without testing its robustness to varying payloads.

In a study conducted by Sheng and Li^30^, the GA (Genetic Algorithm) optimization algorithm was employed to compute the gains of a PID controller for a 3 RRR parallel robot. The objective was to minimize the dynamic error of the system, with the Integral Square Error (ISE) serving as the objective function. The effectiveness of the controller was evaluated under disturbance conditions.

Bounouara et al.^31^ utilized the PSO (Particle Swarm Optimization) optimization algorithm to optimize the PID controller of a two-link manipulator. The Mean Absolute Error (MAE) was employed as the objective function, and the stability of the system was established using the Lyapunov stability theorem. The performance of the controller was evaluated under disturbance conditions, with the disturbances introduced at the measured joint angles.

Loucif et al.^32^ focused on optimizing the PID control of a nonlinear 2-DOF robot manipulator using the Whale Optimizer Algorithm (WOA). The effectiveness of the WOA-PID controller was compared against other controllers such as Particle Swarm Optimization-PID (PSO-PID) and Grey Wolf Optimizer-PID (GWO-PID). The objective function used in this optimization was the Integral Time Absolute Error (ITAE).

The Artificial Bee Colony (ABC) algorithm developed by Karaboga^24, 25^ comprises three types of bees: employed, onlooker, and scout bees. The employed bees actively search for food, while the onlooker bees observe and evaluate the employed bees' dance, which conveys information about food sources. Scout bees are introduced in each generation to maintain solution space diversity and prevent the algorithm from getting trapped in local optima. During their foraging, employed bees gather food and return to the hive, where they perform a waggle dance. This dance contains crucial information such as the direction, distance, and quality rating of flower patches, facilitating effective communication within the colony. By relying solely on the waggle dance, the colony dispatches bees accurately to flower patches without the need for external guides or maps. Each individual bee performs the waggle dance, serving as the primary source of environmental information^28, 33^. The collected food sources are shared among neighboring bees to generate new solutions, which are then evaluated using a fitness function. Additional follower bees are dispatched to promising patches, enabling the colony to efficiently acquire food. If a candidate food source does not lead to improved solutions, it is considered ineffective and replaced^24, 34^.

Similar to other Evolutionary Algorithms (EAs), ABC faces challenges such as early convergence or a sluggish convergence rate when solving complex optimization problems^27, 35, 36^. Research in the ABC community has identified the exploration–exploitation balance as a critical factor affecting performance. The exploration technique equation in the fundamental ABC algorithm exhibits strong performance in searching for new food sources but weak performance during exploitation^37–39^. Thus, enhancing ABC's exploitation while maintaining exploration becomes a significant topic of discussion. Striking a balance between the two is crucial for improving ABC's performance, albeit a challenging task.

The enhanced artificial bee colony with multi-elite guidance (MGABC) is an improved variant of the ABC algorithm introduced by Zhou et al.^39^. This variant incorporates two key modifications aimed at enhancing the exploration and exploitation phases. The first modification focuses on refining the neighborhood search technique, aiming to improve the efficiency of local exploration. This enhancement enables the algorithm to effectively exploit neighboring solutions for potential improvements. The second modification introduces two innovative food search techniques for both exploration and exploitation. These techniques leverage a group of selected superior food sources, referred to as the group of elite solutions. By incorporating the valuable knowledge contained within this group, the algorithm aims to maximize exploitation without compromising exploration. The MGABC algorithm combines these enhancements to achieve a balance between exploration and exploitation, utilizing the valuable insights derived from the group of elite solutions. This approach enhances the algorithm's overall performance and effectiveness in solving optimization problems.

The primary contributions of this research can be summarized as follows:

- Optimization of the PID trajectory tracking controller using the MGABC algorithm.
- Validation of the complex and time-consuming mathematical model through the use of the efficient Simscape model and MSC Adams, providing researchers and industry professionals with a choice between the two approaches.
- Introduction of a novel objective function for the optimization process. This new function is based on the well-known Lyapunov stability functions and is used as an alternative to the widely used functions in the literature.
- Comprehensive performance analysis of the proposed controller, including disturbance rejection at the controller output and robustness against payload uncertainty during pick-and-place operations.
- Investigation of the impact of joint flexibility to evaluate the adaptability of the controller to flexible joint configurations and ensure accurate trajectory tracking.

These contributions provide valuable insights and practical implications for researchers and practitioners working in the field of robotic manipulator systems.

This paper is organized as follows, dynamic models of a 3-DOF planar robotic manipulator using Lagrange formulation, Simscape and MSC Adams software are given in Section "Modelling A 3-DOF robotic manipulator", where the accuracy of the models are verified by investigating the open-loop system responses. Two optimization techniques, the basic Artificial Bee Colony (ABC) and the enhanced Artificial Bee Colony with multi-elite guidance (MGABC), are described in detail in Section "Optimization techniques". The procedures for optimizing the gains of the PID controller for trajectory tracking, utilizing the novel LBF as the objective function are outlined in Section "Optimization of PID gains for trajectory tracking". A comprehensive performance analysis is conducted in Section "Simulation performance evaluation", which includes the elimination of disturbances and the evaluation of robustness against variations in the mass of the end-effector. Additionally, the effect of joint flexibility on the system behavior is discussed. Future research directions are explored in Section "Future work", while concluding remarks summarizing the outcomes of the study are provided in Section "Conclusion".

## Modelling a 3-DOF robotic manipulator

In this section the model of a 3-DOF is formulated using the Lagrangian approach, which accounts for the kinetic and potential energies of the interconnected components. It will be designed to encompass the complexity of the system and its interactions, incorporating relevant variables and assumptions as deemed necessary. As the complexity of a given problem increases, there is a growing demand for variables, assumptions, and iterations, resulting in extended computational time. However, the integration of Simscape Multibody with MATLAB and MSC Adams multibody dynamics and motion analysis software offers engineers the means to examine the dynamics of moving components, the distribution of loads and forces within mechanical systems, and the potential for enhancing and optimizing product performance.

Next subsections will delve into the modeling of the robotic manipulator using Lagrangian approach, Simscape and MSC Adams with a comparative analysis of the open-loop performance of the robotic manipulator using the three different modeling techniques. The internal mechanical properties of the 3DOF manipulator are listed in Table 1.

**Table 1:** The mechanical properties of the links.

Parameters	Link 1	Link 2	Link 3
Mass	1 kg	1 kg	1 kg
Moment of inertia ($${I}_{zz})$$	0.020833 kg m^2^	0.020833 kg m^2^	0.020833 kg m^2^
Length	0.5 m	0.5 m	0.5 m
Cross-section area	5 × 5 cm^2^	5 × 5 cm^2^	5 × 5 cm^2^

### Lagrangian mathematical modelling

**Figure 1:**
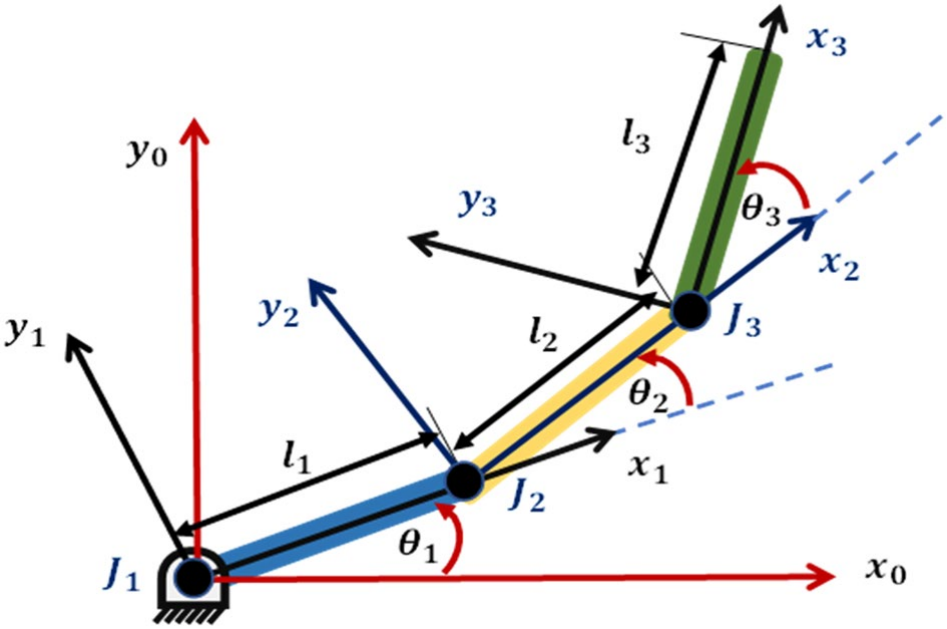
Schematic diagram of the 3-DOF robotic model.

Figure 1 presents the schematic representation of a 3DOF rigid planar robotic manipulator model. In this diagram, the initial link is fixed to a rigid base through a pin support that operates without friction. Subsequently, the second link is positioned at the extremity of the first link and secured by a frictionless ball bearing. Similarly, the third link is connected to the second link through another frictionless ball bearing. The system's non-linear coupled dynamic equations are derived and expressed in Eq. (1).

1 $$\left[\begin{array}{ccc}{M}_{11}& {M}_{12}& {M}_{13}\\ {M}_{21}& {M}_{22}& {M}_{23}\\ {M}_{31}& {M}_{32}& {M}_{33}\end{array}\right]\left[\begin{array}{c}{\ddot{\theta }}_{1}\\ {\ddot{\theta }}_{2}\\ {\ddot{\theta }}_{3}\end{array}\right]+\left[\begin{array}{c}{F}_{1}\left(\theta ,\dot{\theta }\right)\\ {F}_{2}\left(\theta ,\dot{\theta }\right)\\ {F}_{3}\left(\theta ,\dot{\theta }\right)\end{array}\right]+\left[\begin{array}{c}{g}_{1}\left(\theta \right)\\ {g}_{2}\left(\theta \right)\\ {g}_{3}\left(\theta \right)\end{array}\right]=\left[\begin{array}{c}{\tau }_{1}\\ {\tau }_{2}\\ {\tau }_{3}\end{array}\right]$$

where ${\ddot{\theta }}_{i}$ , ${F}_{i}\left(\theta ,\dot{\theta }\right) , {g}_{i}$ and ${\tau }_{i}$ for ($i=\mathrm{1,2} ,3$) represent the angular accelerations of the three links, the combined centrifugal and Coriolis forces, the gravitational forces, and the torque exerted at each joint of the three links, respectively. The lefthand matrix defined by ${M}_{ii}$ is the robot mass matrix and its entries are defined by:

2 $${M}_{11}={{l}_{1}}^{2}\left(0.25{m}_{1}+{m}_{2}+{m}_{3}\right)+{{l}_{2}}^{2}\left(0.25{m}_{2}+{m}_{3}\right)+0.25{{l}_{3}}^{2}{m}_{3}+{l}_{1}{l}_{2}cos\left({\theta }_{2}\right)\left({m}_{2}+2{m}_{3}\right)+{l}_{2}{l}_{3}{m}_{3} cos\left({\theta }_{3}\right)+{l}_{1}{l}_{3}{m}_{3}cos\left({\theta }_{2}+{\theta }_{3}\right)+{I}_{1}+{I}_{2}+{I}_{3}$$

3 $${M}_{12}={{l}_{2}}^{2}\left(0.25{m}_{2}+{m}_{3}\right)+0.25{{l}_{3}}^{2}{m}_{3}+{l}_{1}{l}_{2} cos\left({\theta }_{2}\right)\left({m}_{3}+0.5{m}_{2}\right) +{l}_{2}{l}_{3} {m}_{3} cos\left({\theta }_{3}\right)+0.5 {l}_{1}{l}_{3} {m}_{3} cos\left({\theta }_{2}+ {\theta }_{3}\right) +{I}_{2}+{I}_{3}$$

4 $${M}_{13}=0.25{{l}_{3}}^{2}{m}_{3}+0.5{l}_{2}{l}_{3} {m}_{3} cos\left({\theta }_{3}\right) +0.5{l}_{1}{l}_{3}{m}_{3} cos\left({\theta }_{2}+ {\theta }_{3}\right)+{I}_{3}$$

5 $${M}_{21}={{l}_{2}}^{2}\left(0.25{m}_{2}+{m}_{3}\right)+0.25{{l}_{3}}^{2}{m}_{3}+{l}_{1}{l}_{2}cos\left({\theta }_{2}\right)\left({m}_{3}+0.5{m}_{2}\right) +{l}_{2}{l}_{3}{m}_{3} cos\left({\theta }_{3}\right)+0.5{l}_{1}{l}_{3}{m}_{3} cos\left({\theta }_{2}+ {\theta }_{3}\right)+{I}_{2}+{I}_{3}$$

6 $${M}_{22}={{l}_{2}}^{2}\left(0.25{m}_{2}+{m}_{3}\right)+0.25{{l}_{3}}^{2}{m}_{3}+{l}_{2}{l}_{3}{m}_{3}cos\left({\theta }_{3}\right)+{I}_{2}+{I}_{3}$$

7 $${M}_{23}=0.25{{l}_{3}}^{2}{m}_{3}+0.5{l}_{2}{l}_{3}{m}_{3}cos\left({\theta }_{3}\right)+{I}_{3}$$

8 $${M}_{31}=0.25{{l}_{3}}^{2}{m}_{3}+0.5{l}_{2}{l}_{3}{m}_{3} cos\left({\theta }_{3}\right) +0.5{l}_{1}{l}_{3}{m}_{3} cos\left({\theta }_{2}+ {\theta }_{3}\right)+{I}_{3}$$

9 $${M}_{32}=0.25{{l}_{3}}^{2}{m}_{3}+0.5{l}_{2}{l}_{3}{m}_{3}cos\left({\theta }_{3}\right) +{I}_{3}$$

10 $${M}_{33}=0.25{{l}_{3}}^{2}{m}_{3}+{I}_{3}$$

where, ${m}_{i}$, ${l}_{i}$ and ${I}_{i}$ represent the mass, length, and the moment of inertia of the ith link about its center of gravity.

The various centrifugal and Coriolis forces are given as follows:

11 $${F}_{1}\left(\theta ,\dot{\theta }\right)={-\left({\dot{\theta }}_{2}\right)}^{2}\left(0.5{l}_{1}{l}_{2}{m}_{2} sin\left({\theta }_{2}\right)+{l}_{1}{l}_{2}{m}_{3} sin\left({\theta }_{2}\right)+0.5{l}_{1}{l}_{3}{m}_{3} sin\left({\theta }_{2}+ {\theta }_{3}\right) \right)-{{(\dot{\theta }}_{3})}^{2}\left(0.5{l}_{2}{l}_{3}{m}_{3} sin\left({\theta }_{3}\right)+0.5{l}_{1}{l}_{3}{m}_{3} sin\left({\theta }_{2}+ {\theta }_{3}\right)\right)-{\dot{\theta }}_{1}{\dot{\theta }}_{2}\left({l}_{1}{l}_{2}{m}_{2} sin\left({\theta }_{2}\right)+2{l}_{1}{l}_{2}{m}_{3} sin\left({\theta }_{2}\right)+{l}_{1}{l}_{3} {m}_{3} sin\left({\theta }_{2}+{\theta }_{3}\right)\right)-{\dot{\theta }}_{2}{\dot{\theta }}_{3}\left({l}_{1}{l}_{3}{m}_{3} sin\left({\theta }_{2}+ {\theta }_{3}\right)+{l}_{2}{l}_{3}{m}_{3} sin\left({\theta }_{3}\right)\right)-{\dot{\theta }}_{1}{\dot{\theta }}_{3}\left({l}_{2}{l}_{3}{m}_{3} sin\left({\theta }_{3}\right)+{l}_{1}{l}_{3}{m}_{3} sin\left({\theta }_{2}+{\theta }_{3}\right)\right)$$

12 $${F}_{2}\left(\theta ,\dot{\theta }\right)={\left({\dot{\theta }}_{1}\right)}^{2}\left(0.5{l}_{1}{l}_{2}{m}_{2} sin\left({\theta }_{2}\right)+{l}_{1}{l}_{2}{m}_{3} sin\left({\theta }_{2}\right)+0.5{l}_{1}{l}_{3}{m}_{3}sin\left({\theta }_{2}+{\theta }_{3}\right) \right)-0.5{{(\dot{\theta }}_{3})}^{2}\left({l}_{2}{l}_{3}{m}_{3}sin\left({\theta }_{3}\right)\right)-{\dot{\theta }}_{1}{\dot{\theta }}_{3}\left( {l}_{2}{l}_{3}{m}_{3}sin\left({\theta }_{3}\right)\right)-{\dot{\theta }}_{2}{\dot{\theta }}_{3}\left({l}_{2}{l}_{3}{m}_{3}sin\left({\theta }_{3}\right)\right)$$

13 $${F}_{3}\left(\theta ,\dot{\theta }\right)={\left({\dot{\theta }}_{1}\right)}^{2}\left(0.5{l}_{2}{l}_{3}{m}_{3}sin\left({\theta }_{3}\right)+0.5{l}_{1}{l}_{3}{m}_{3}sin\left({\theta }_{2}+{\theta }_{3}\right)\right)+0.5{\left({\dot{\theta }}_{2}\right)}^{2}\left({l}_{2}{l}_{3}{m}_{3}sin\left({\theta }_{3}\right)\right)+{\dot{\theta }}_{1}{\dot{\theta }}_{2}\left({l}_{2}{l}_{3}{m}_{3}sin\left({\theta }_{3}\right)\right)$$

The gravitational forces are defined by:

14 $${g}_{1}\left(\theta \right)={m}_{3}g\left({l}_{1}cos\left({\theta }_{1}\right)+0.5{l}_{3}cos\left({\theta }_{1}+{\theta }_{2}+ {\theta }_{3}\right) +{l}_{2} cos\left({\theta }_{1}+ {\theta }_{2}\right)\right)+ {m}_{2}g\left({l}_{1} cos\left({\theta }_{1}\right)+0.5{l}_{2} cos\left({\theta }_{1}+ {\theta }_{2}\right)\right)+0.5{m}_{1}g l1 cos\left({\theta }_{1}\right)$$

15 $${g}_{2}\left(\theta \right)={m}_{3}g\left(0.5{l}_{3}cos\left({\theta }_{1}+{\theta }_{2}+{\theta }_{3}\right) +{l}_{2} cos\left({\theta }_{1}+ {\theta }_{2}\right)\right)+0.5{m}_{2}g {l}_{2} cos\left({\theta }_{1}+{\theta }_{2}\right)$$

16 $${g}_{3}\left(\theta \right)=0.5{m}_{3}g{l}_{3}cos\left({\theta }_{1}+{\theta }_{2}+{\theta }_{3}\right)$$

where, $g$ represents the acceleration of gravity.

### Simscape model

The Simulink® environment provides a platform for constructing physical model systems efficiently. Simscape™, within this environment, facilitates the creation of physical component models that are established on interconnected physical connections and seamlessly interact with block diagrams and other modeling methodologies. By incorporating Simscape add-on products, users gain access to advanced components and analysis tools, further enhancing the modeling capabilities. Simscape significantly contributes to the development of control systems and the assessment of system performance. Leveraging MATLAB variables and expressions, models can be parameterized, while Simulink serves as a valuable tool for devising control strategies for physical systems^2, 11^.

**Figure 2:**
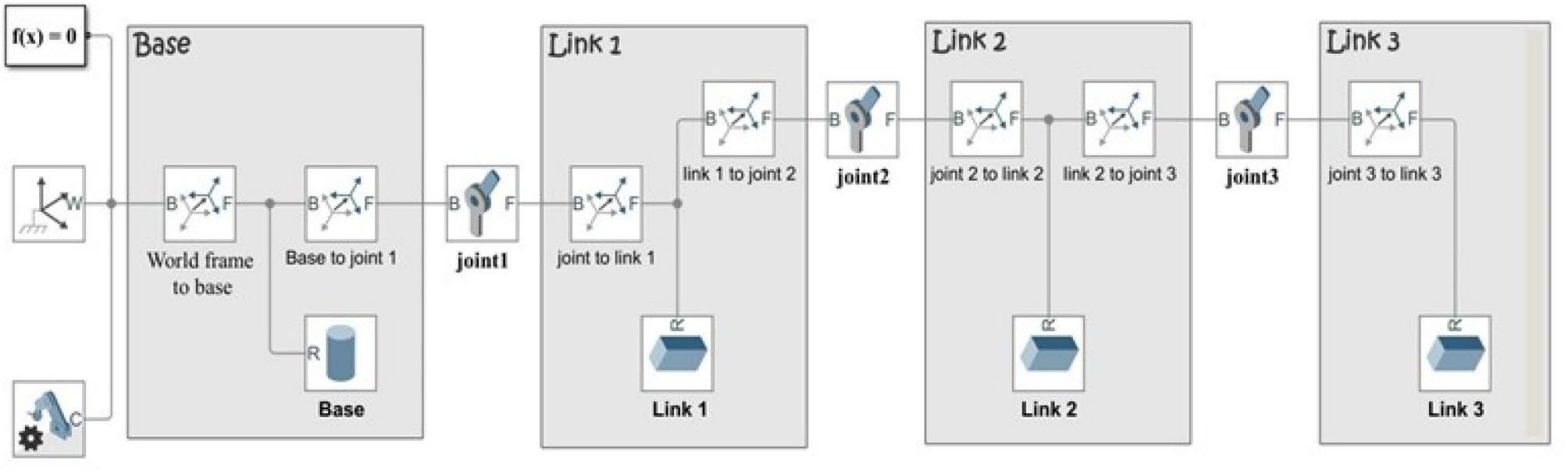
Simscape model of the 3-DOF robotic manipulator.

Figure 2 illustrates the Simscape model of the RLM system. The model encompasses all the essential mechanical attributes of the components, generated by the MATLAB SimMechanics Toolbox.

## MSC Adams model

MSC Adams is a widely used software tool for modeling and simulating the dynamics of robotic manipulators. It is specifically designed for multibody dynamics analysis, which allows engineers to study the motion and behavior of interconnected mechanical systems. It considers the interactions between different components, such as links, joints, and actuators, allowing for a comprehensive analysis of the system's behavior. The software provides tools for studying the kinematics and dynamics of the robotic manipulator.

**Figure 3:**
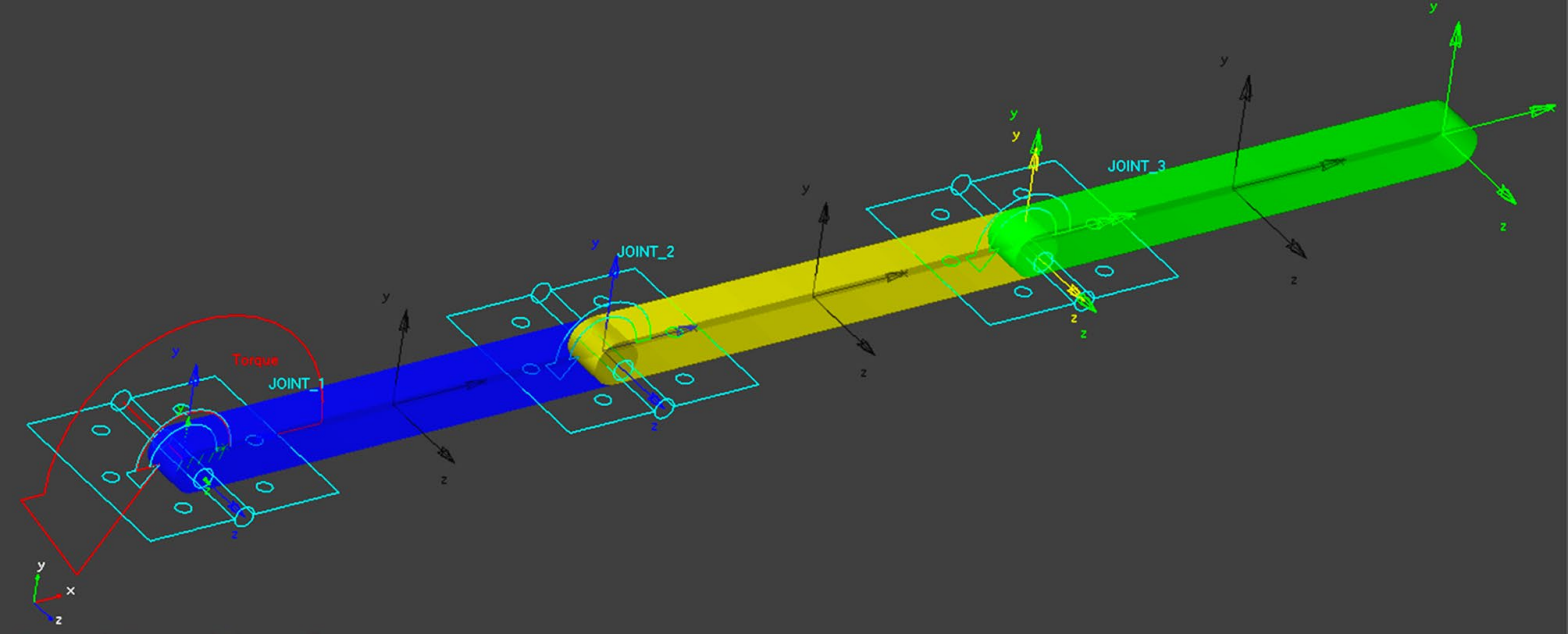
MSC Adams model of the 3-DOF robotic manipulator.

This enables engineers to understand how the manipulator moves and responds to external loads or control inputs. MSC Adams can be integrated with control systems and algorithms developed in other software tools, such as MATLAB and Simulink. This integration enables engineers to design and evaluate control strategies for the robotic manipulator within a unified simulation environment. The MSC Adams model of the RLM system is depicted in Fig. 3.

### Validation of the modeling techniques

**Figure 4:**
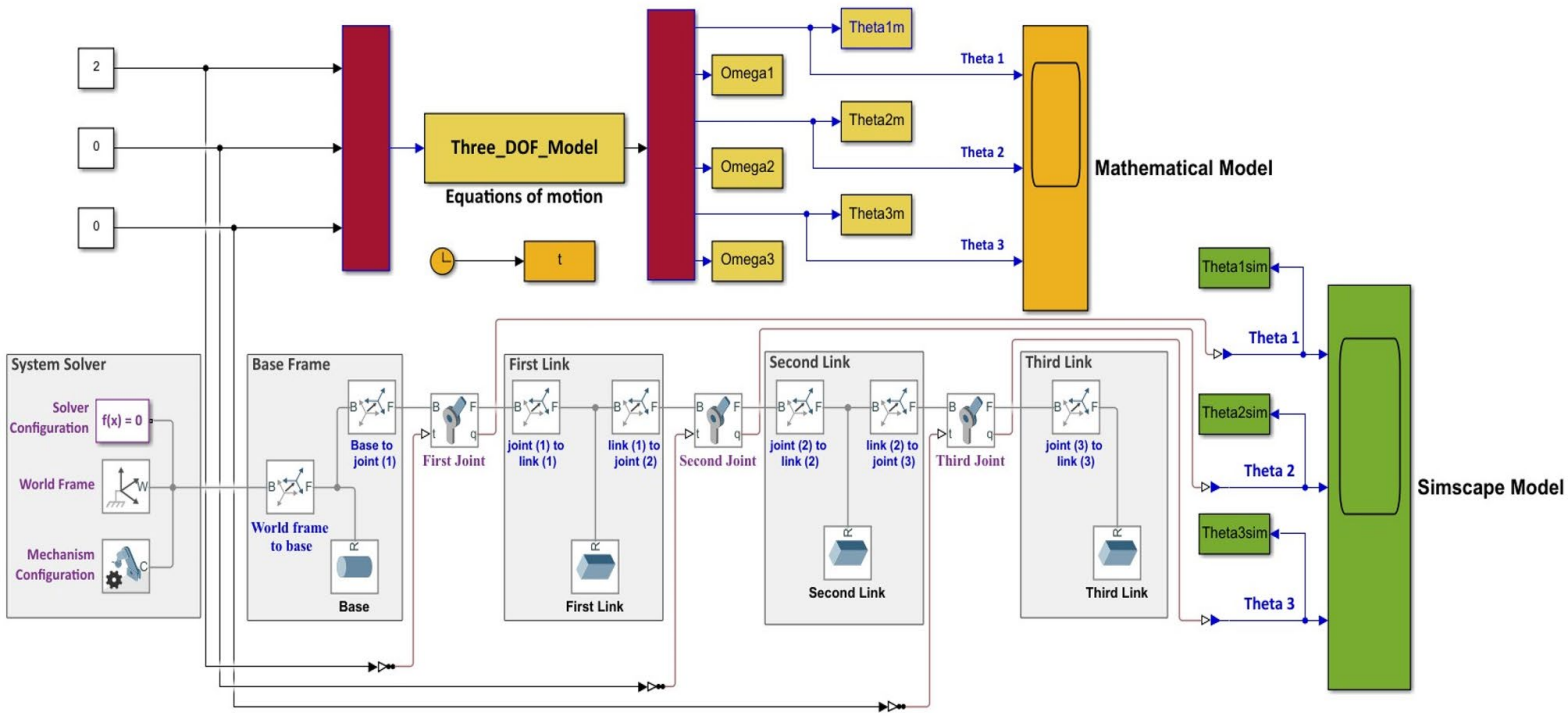
System's configuration during the validation of the mathematical and the Simscape models.

In order to validate the modeling techniques employed, a comparison is conducted, focusing on the open loop performance of three distinct models: the mathematical model, the Simscape model, and the MSC Adams model. This validation analysis is performed under the condition wherein a constant torque with a magnitude of 2 N m is applied to the first joint of the robotic manipulator. Figures 3 and 4 visually illustrate the system's configuration during this validation procedure.

**Figure 5:**
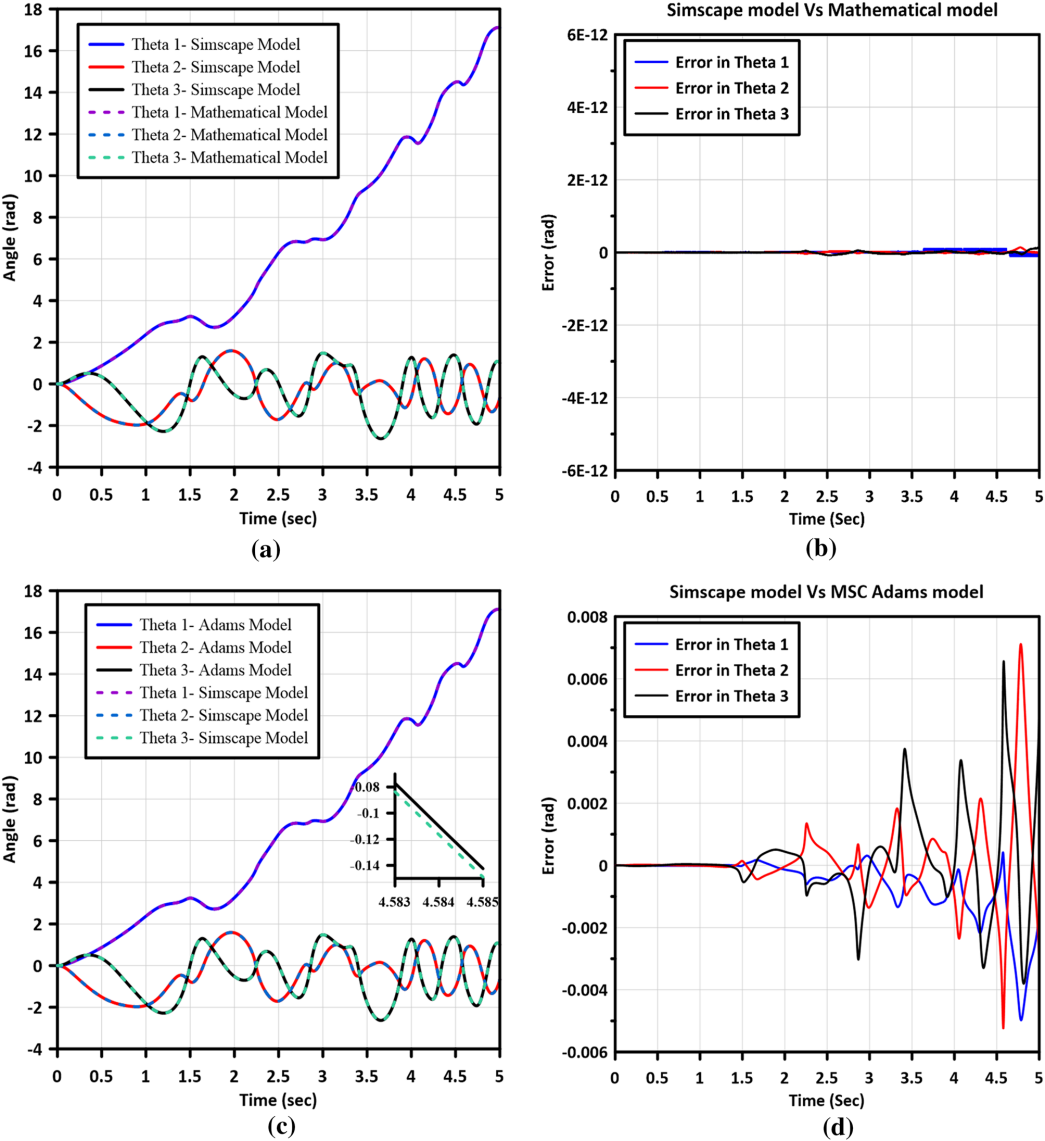
Model validation results, (**a**) the response of the Simscape and Mathematical models, (**b**) the error between Simscape and Mathematical models, (**c**) the response of the Simscape and MSC Adams models, and (**d**) the error between the Simscape and MSC Adams models.

Figure 5 presents the outcomes derived from the comparative analysis of the three modeling techniques. Figure 5a,b exhibit the response of the mathematical and Simscape models, demonstrating their remarkable similarity, with an error in angles equal to zero. Similarly, Fig. 5c,d exhibit the response of the MSC Adams and Simscape models. Notably, the models demonstrate substantial similarity, with the error in angles bounded within the range of $-0.006$ to $0.006$, which is deemed negligible.

Considering the close resemblance and insignificant differences among the three proposed techniques, employing the Simscape Multibody toolbox is recommended for modeling multi-Degree-of-Freedom (MDOF) robotic manipulator systems. This preference arises from the advantages offered by Simscape, such as rapid modeling capabilities and ease of incorporating and modifying components, which are comparatively more challenging to achieve using MSC Adams software.

Our approach offers distinct advantages over the methodologies employed by Lee et al.^11^ and Manjaree and Thomas^12^. Unlike their approaches, our methodology does not require the use of CAD software such as Solidworks. Additionally, our study places emphasis on validating mathematical model against models created using software tools like MATLAB and MSC Adams. This aspect has not been adequately addressed in previous research, making our investigation particularly valuable for researchers and practitioners working in the field of robotic manipulator systems. Furthermore, our approach is flexible and can be extended to systems with higher degrees of freedom. With appropriate modifications and considerations to account for the increased complexity, our methodology can be applied effectively. The ability to apply our approach to higher degree-of-freedom systems opens up new possibilities and expands the scope of its practical applications. Overall, our research contributes valuable insights and offers practical implications for the development and analysis of robotic manipulator systems.

## Optimization techniques

The subsequent subsections provide a comprehensive exposition of the basic artificial bee colony (ABC)^25^ and enhanced artificial bee colony with multi-elite guidance (MGABC)^39^ optimization techniques, elucidating their intricate mechanisms and algorithms.

### The Basic ABC Algorithm

The basic artificial bee colony (ABC) algorithm simulates a honeybee colony consisting of three distinct types of bees: employed bees, onlooker bees, and scout bees. Each type of bee is assigned specific tasks within the optimization process. The employed bees are responsible for exploring the search area to discover nutritious food sources. They actively search for potential solutions, acting as the exploratory agents in the algorithm. It is important to note that the number of employed bees is equivalent to the number of food sources within the search area. To expedite the search process in subsequent iterations, the employed bees share information about the quality and distance to the food source with the onlooker bees at the hive. This information exchange involves communicating the richness of nectar (representing the fitness value of a potential solution) associated with each food source. The onlooker bees then utilize the acquired information to selectively explore the neighborhood of chosen food sources. Food sources with higher fitness values have a greater probability of being selected by the onlooker bees. This exploitation mechanism enhances the algorithm's ability to refine and improve promising solutions. The number of onlooker bees is equal to the number of employed bees, ensuring a balanced distribution of exploration and exploitation strategies in the ABC algorithm. These collaborative efforts among the employed and onlooker bees contribute to the overall optimization process, enabling efficient search and convergence towards optimal solutions^25, 27, 36, 39, 40^.

Scouter bees are added in each generation to ensure the diversity of the solution space and prevent the algorithm from being stuck in a local optimum. A food source is abandoned if it cannot be improved for more than a limited number of trials. In turn, the employed bee connected to the discarded solution will change into a scouter bee and begin searching for a new food source throughout the entire search area. The Algorithm procedures can be described with four stages as following:

#### Initialization stage

In this stage the ABC starts with initial set of $SN$ food sources which represents the possible solutions. The initial values of an individual ${X}_{k} =({x}_{k,1}, {x}_{k,2},\ldots, {x}_{k,S})$ are generated using Eq. (17)^24, 25^ which represents the number of food sources (candidate solutions), where $S$ denotes the dimension size of the parameters to be optimized.

17 $${x}_{k,j}={{x}_{j}}_{min}+rand\left(\mathrm{0,1}\right)\times \left({{x}_{j}}_{max}-{{x}_{j}}_{min}\right)$$

where $k \in (1,2,\dots ,SN)$ and $j\in (\mathrm{1,2},\dots ,S)$. ${{x}_{j}}_{min}$ and ${{x}_{j}}_{max}$ are the lower and upper bounds of the $Sth$ dimensions, respectively.

#### Exploration stage

The employed bees will explore new food sources throughout the search area, and new individuals in the subsequent search strategy are generated utilizing the exploration search equation specified in Eq. (18)^24, 25^.

18 $${v}_{k,j}={x}_{k,j}+{\phi }_{k,j}\times \left({x}_{k,j}-{x}_{p,j}\right)$$

where ${v}_{k,j}$ is the new source of food. ${x}_{p,j}$ is a partner food source chosen at random from the population, and it must be dissimilar from ${x}_{k,j}$. $\phi$ is a uniformly random number between $[-1, 1]$^27, 39^. It should be noted that only one dimension of ${X}_{k}$ is altered to produce ${V}_{k}$.

If ${v}_{k,j}$ exceeds the upper bound, it will be reset to the upper value. Conversely, if it falls below the lower value, it will be reset to the lower value. The selection of the best solution involves comparing the fitness values of the previous and current solutions and employing a greedy selection approach. Depending on the fitness value, the counter associated with ${X}_{k}$ is either reset to 0 or incremented by 1^41^. It is important to note that each food source has a counter limit, which keeps track of the consecutive iterations where it has not been improved^39^.

#### Exploitation stage

The employer bees and the onlooker bees both have the same process of exploitation. The main distinction between them is the choosing of promising food sources based on the probabilities determined by the fitness values. Depending on the information the explorer bees collect (i.e., quality, amount, distance between the food source and the hive, etc.), a particular food source may be chosen more often. High probability indicates the presence of a significant quantity of excellent nectar. It should be mentioned that the onlooker bees only explore around the specified food sources' neighborhood. The possibility of being chosen for a promising nectar source can be calculated by Eq. (19)^24, 25^

19 $${p}_{k}= \frac{{fit}_{k}}{\sum_{k=1}^{SN}{fit}_{k}}$$

where ${fit}_{k}$ is the fitness value of solution $k$, and SN is the number of employed bees or food sources. The fitness value ${fit}_{k}$ of each food source is calculated using Eq. (20).

20 $${fit}_{k}=\left\{\begin{array}{cc}\frac{1}{1+f({X}_{k})}& if f\left({X}_{k}\right)\ge 0\\ 1+\left|f({X}_{k})\right|& otherwise\end{array}\right\}$$

where $f({X}_{k})$ is the value of the objective function. A food source is considered to have been abandoned if it cannot be further developed in a preset number of loops; however, if a new food source is superior to its parent, it will be kept, and the related counter will be reset to 0^25, 36, 42^.

#### Scouter stage

For every nectar source, a check will be made on the corresponding counter of abandonment, in the ABC algorithm, the value of the specified number of loops is a crucial control parameter known as the abandonment limit. If ${X}_{k}$ represents the abandoned food source, then the scout bee generates a new random food source according to Eq. (17).

### The enhanced ABC algorithm

Zhou et al.^39^ proposed two modified solution search equations in their study. These equations are specifically designed for the exploration stage and the exploitation stage. Although the exploration stage and the exploitation stage in the basic Artificial Bee Colony (ABC) algorithm share the same exploration search equation to generate new offspring, the roles of employed bees and onlooker bees are different within the internal mechanism of ABC^36, 39^. Therefore, it is recommended to develop separate solution search equations for the employed bee phase and the onlooker bee phase. The modified algorithm procedures of the Multi-Elite Guidance Artificial Bee Colony (MGABC) can be outlined as follows:

#### Initialization stage

The initialization stage in the MGABC is the same as that of the standard ABC.

#### Exploration stage

Since employed bees are in charge of discovering new solutions across the whole search space, the employed bee phase's solution search equation should maintain relatively robust exploration. As a result, the used bee phase uses the modified solution search equation mentioned in Eq. (21)^43^.

21 $${v}_{k,j}={x}_{{r}_{1},j}+{\phi }_{k,j}\times \left({x}_{{r}_{1},j}-{x}_{{r}_{2},j}\right)$$

where ${X}_{r1}$ and ${X}_{r2}$ are two distinct food sources that were chosen at random from the population and are both distinct from ${X}_{k}$. In the range of $[-\mathrm{1,1}]$, $\phi$ is a uniformly distributed random number. The food sources used in Eq. (21) to generate candidate solutions are all chosen randomly from the population.

#### Exploitation stage

Unlike the exploration phase, the onlooker bees are primarily focused on exploitation in order to conduct thorough searches for favorable food sources in the vicinity, resulting in the production of new offspring. As a result, an innovative solution search technique utilizing multiple elite solutions has been formulated specifically for the onlooker bees, and it is represented by Eq. (22)^39^.

22 $${v}_{k,j}=\left\{\begin{array}{cc}{x}_{e,j}+{\phi }_{k,j}\times \left({x}_{e,j}-{x}_{k,j}\right)& if rand(\mathrm{0,1})\le MR\\ {x}_{k,j}& otherwise\end{array}\right\}$$

where ${X}_{e}$ is one of the most promising solutions from the current population that was randomly chosen from the elite group. $Se=q.SN$ denotes the size of the elite group. The purpose of the control parameter $MR$ is to regulate how many dimensions can be sent from the superior solution ${X}_{e}$ to the new solution ${V}_{k}$^41^.

#### Scouter stage

The scout stage in the MGABC is the same as that of the standard ABC.

#### Modified neighborhood search operator stage

The basic principle of the modified neighbourhood search operator is that, like other evolutionary algorithms, ABC also frequently shows an unsatisfactory performance when solving challenging issues like premature convergence and sluggish convergence pace. These could be caused, for example, by an excessively large search step size that makes it likely that the true solution would be missed^26, 27, 40, 41^. The operator thus performs particularly good exploitation and has a simple structure, which can be considered as a local search tool after the main ABC procedure is listed in Eq. (23)^39^, and if one solution is unfortunate became stuck by one of the local optima, exploring the neighbourhoods of this solution can help locate better alternatives or even the best solution^39^.

23 $${TX}_{k}={r}_{1}\cdot {X}_{k}+{r}_{2}\cdot {X}_{e1}+{r}_{3}\cdot \left({X}_{e2}-{X}_{e3}\right)$$

where ${X}_{e1}$, ${X}_{e2}$, and ${X}_{e3}$ are the three food sources chosen at random from the elite group, and they must be distinct from ${X}_{k}$. Note that the group of superior food sources utilised in Eqs. (22) and (23) is the same.

## Optimization of PID gains for trajectory tracking

On a personal computer with an Intel(R) Core (TM) i7-10750H CPU running at 2.60 GHz, 16 GB of RAM, and a 64-bit operating system, all the simulations provided here were run in MATLAB/SIMULINK. The ODE solver used a fourth-order Runge–Kutta method with 0.001 s sample time. The torque limitations used for all links are $[-200, 200]$ N.m. For links 1, 2, and 3, the desired trajectories (${\theta }_{d1}$, ${\theta }_{d2}$, and ${\theta }_{d3}$) have been listed in Eqs. (25), (26) and (27), respectively. Table 2 lists the various ABC and MGABC parameters that are utilised to maximize controller gains. The simulation time is taken to be 5 s.

**Table 2:** Design parameters of ABC and MGABC.

ABC		MGABC	
Design parameter	Value	Design parameter	Value
Colony size ($N$)	20	Colony size ($N$)	20
Lower bound ($LB$)	[300 100 15]	Lower bound ($LB$)	[300 100 15]
Upper bound ($UB$)	[550 200 65]	Upper bound ($UB$)	[550 200 65]
Iterations ($Itr$)	40	Iterations ($Itr$)	40
		Modification rate ($MR$)	0.5
		Elite group ($Se$)	3

**Figure 6:**
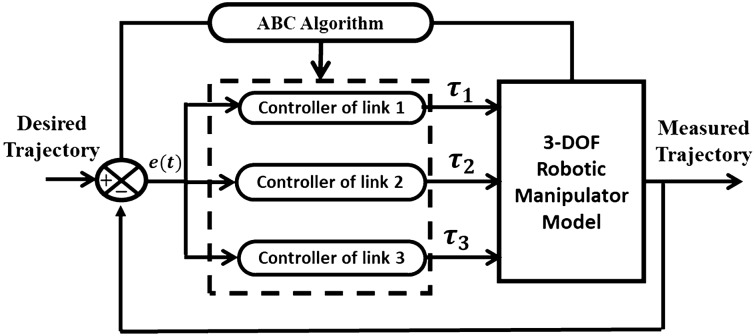
Schematic diagram of robotic system PID tuning.

A fundamental ABC-PID tuning scheme is shown in Fig. 6. PID controller determines the control signal that activates the actual system according to Eq. (24).

24 $$\tau \left(t\right)={K}_{p}{e}_{\theta }\left(t\right)+{K}_{I}\int e\left(t\right)dt+{K}_{d}\frac{d{e}_{\theta }\left(t\right)}{dt}$$

where the controller parameter is $\tau \left(t\right)$ which considered to be the torque applied at each joint, the error is ${e}_{\theta }\left(t\right)$ which is the difference between the desired ${\theta }_{d}\left(t\right)$ and measured ${\theta }_{m}\left(t\right)$ signals, respectively. The controller parameters that need to be tuned are ${K}_{p}$*,* ${K}_{I}$, and ${K}_{d}$. Computer simulations have produced numerical results that have been used to evaluate the capabilities of the suggested tuning methods.

25 $${\theta }_{d1}=\mathrm{sin}\left(2t\right)$$

26 $${\theta }_{d2}=\mathrm{sin}\left(2t\right)$$

27 $${\theta }_{d3}=\mathrm{cos}\left(2t\right)$$

A newly proposed Lyapunov-based function (LBF), as denoted by Eq. (28), was utilized as the objective function during the tuning process. This new function was compared against five error-based objective functions mentioned in the existing literature. These objective functions include the Integral Time Absolute Error (ITAE) described in Eq. (29), Integral Absolute Error (IAE) defined in Eq. (30), Integral Square Error (ISE) presented in Eq. (31), Mean Root Square Error (MRSE) outlined in Eq. (32) and Mean Absolute Error (MAE) specified in Eq. (33). The purpose of this comparison was to evaluate the effectiveness and performance of the novel LBF relative to the established error-based objective functions.

28 $$LBF=\sum_{i=1}^{3}\sum_{k=1}^{N}{\left[\begin{array}{c}{e}_{{\theta }_{i}}(k)\\ {e}_{{d}_{i}}(k)\\ {e}_{{v}_{i}}(k)\end{array}\right]}^{T}\left[\begin{array}{ccc}\alpha & 0& 0\\ 0& \beta & 0\\ 0& 0& \gamma \end{array}\right]\left[\begin{array}{c}{e}_{{\theta }_{i}}(k)\\ {e}_{{d}_{i}}(k)\\ {e}_{{v}_{i}}(k)\end{array}\right]$$

29 $$ITAE ={\int }_{0}^{t}t\left|e(t)\right|dt$$

30 $$IAE ={\int }_{0}^{t}\left|e(t)\right|dt$$

31 $$ISE ={\int }_{0}^{t}e{\left(t\right)}^{2}dt$$

32 $$MRSE =\frac{1}{N}\sum_{k=1}^{N}\sqrt{{e}_{{\theta }_{1}}{\left(k\right)}^{2}+{e}_{{\theta }_{2}}{\left(k\right)}^{2}+{e}_{{\theta }_{3}}{\left(k\right)}^{2}}$$

33 $$MAE =\frac{1}{N}\sum_{k=1}^{N}\left|{e}_{{\theta }_{1}}(k)\right|+\left|{e}_{{\theta }_{2}}(k)\right|+\left|{e}_{{\theta }_{3}}(k)\right|$$

34 $${e}_{{\theta }_{i}}\left(k\right)={\theta }_{{d}_{i}}\left(k\right)-{\theta }_{{m}_{i}}\left(k\right)$$

35 $${e}_{{d}_{i}}(k)=\frac{d{e}_{{\theta }_{i}}\left(k\right)}{dt}$$

36 $${e}_{{v}_{i}}(k)={\int }_{0}^{t}{e}_{{\theta }_{i}}\left(k\right) dt$$

The terms ${e}_{{\theta }_{i}}(t)$ , ${e}_{{d}_{i}}(k)$ and ${e}_{{v}_{i}}(k)$ represent the errors, derivative of errors, and integral of errors in the measured angle of link i, where i corresponds to the number of links (i.e., $i = 1, 2, 3$). The constants in Eq. (28) are chosen to be $\alpha =0.7$, $\beta =0.2$ and $\gamma =0.1$. Table 3 presents the optimized gains of the controller for all three links, utilizing the LBF as well as the other functions mentioned in previous literature. It is noteworthy that the optimal gains remained unchanged throughout the entire study.

**Table 3:** Optimized gains of PID Controller. Significant values are in bold.

Objective functions	Algorithm	Optimized gains								
		Link (1)			Link (2)			Link (3)		
		${K}_{p}$	${K}_{i}$	${K}_{d}$	${K}_{p}$	${K}_{i}$	${K}_{d}$	${K}_{p}$	${K}_{i}$	${K}_{d}$
LBF	ABC	550.000	135.645	65.000	550.000	101.398	63.957	550.000	100.000	59.088
	**MGABC**	**550.000**	**104.679**	**65.000**	**550.000**	**200.000**	**65.000**	**550.000**	**136.745**	**55.056**
ITAE^23, 32, 44^	ABC	534.161	100.000	64.039	550.000	135.418	65.000	508.234	200.000	23.2699
	MGABC	550.000	100.216	65.000	550.000	102.799	15.000	550.000	195.288	15.000
IAE^15^	ABC	550.000	100.00	65.000	550.000	100.000	65.000	525.998	196.129	65.000
	MGABC	550.000	100.000	65.000	550.000	100.000	65.000	550.000	100.168	65.000
ISE^30^	ABC	550.000	131.742	65.000	550.000	117.990	65.000	550.000	100.000	65.000
	MGABC	550.000	100.000	65.000	550.000	153.059	65.000	550.000	196.701	65.000
MRSE^29^	ABC	550.000	101.249	65.000	539.630	100.215	58.284	550.000	200.000	26.477
	MGABC	550.000	100.00	65.000	550.000	100.000	65.000	550.000	200.000	22.956
MAE^29, 31^	ABC	550.000	100.00	64.315	530.912	100.000	65.000	387.838	189.629	15.000
	MGABC	550.000	100.00	65.000	550.000	100.000	65.000	427.911	200.000	15.000

**Figure 7:**
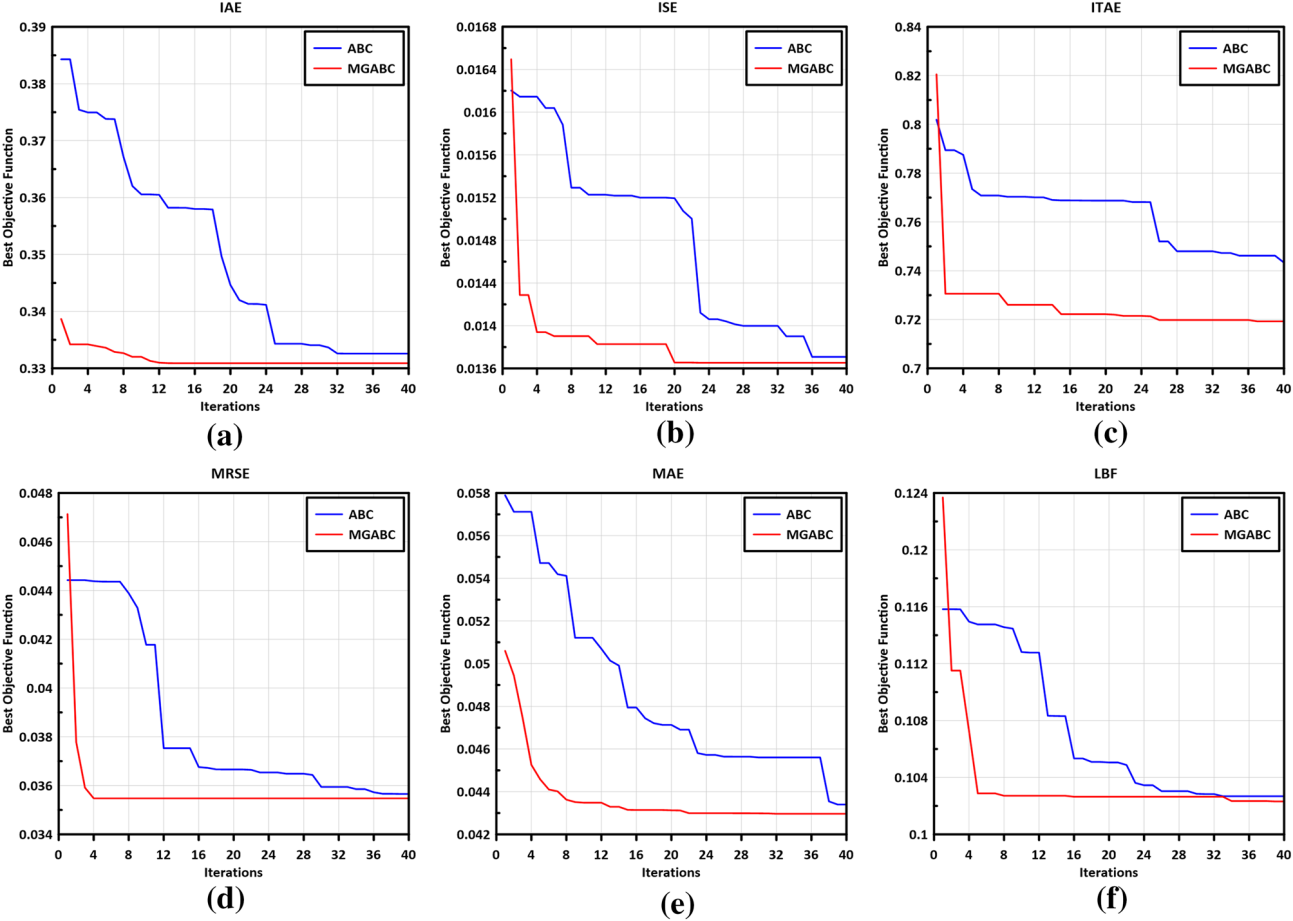
Convergence history of (**a**) IAE, (**b**) ISE, (**c**) ITAE, (**d**) MRESE, (**e**) MAE and (**f**) LBF.

Figure 7 shows the curves of the objective functions (OFs) versus iteration for all tested objective functions, employing two optimization algorithms. It is evident from Fig. 7 that the MGABC achieved lower OF values compared to the ABC algorithm. This can be attributed to the MGABC's effective exploration of the search space and its utilization of various exploitation schemes, enabling it to avoid local optima.

**Figure 8:**
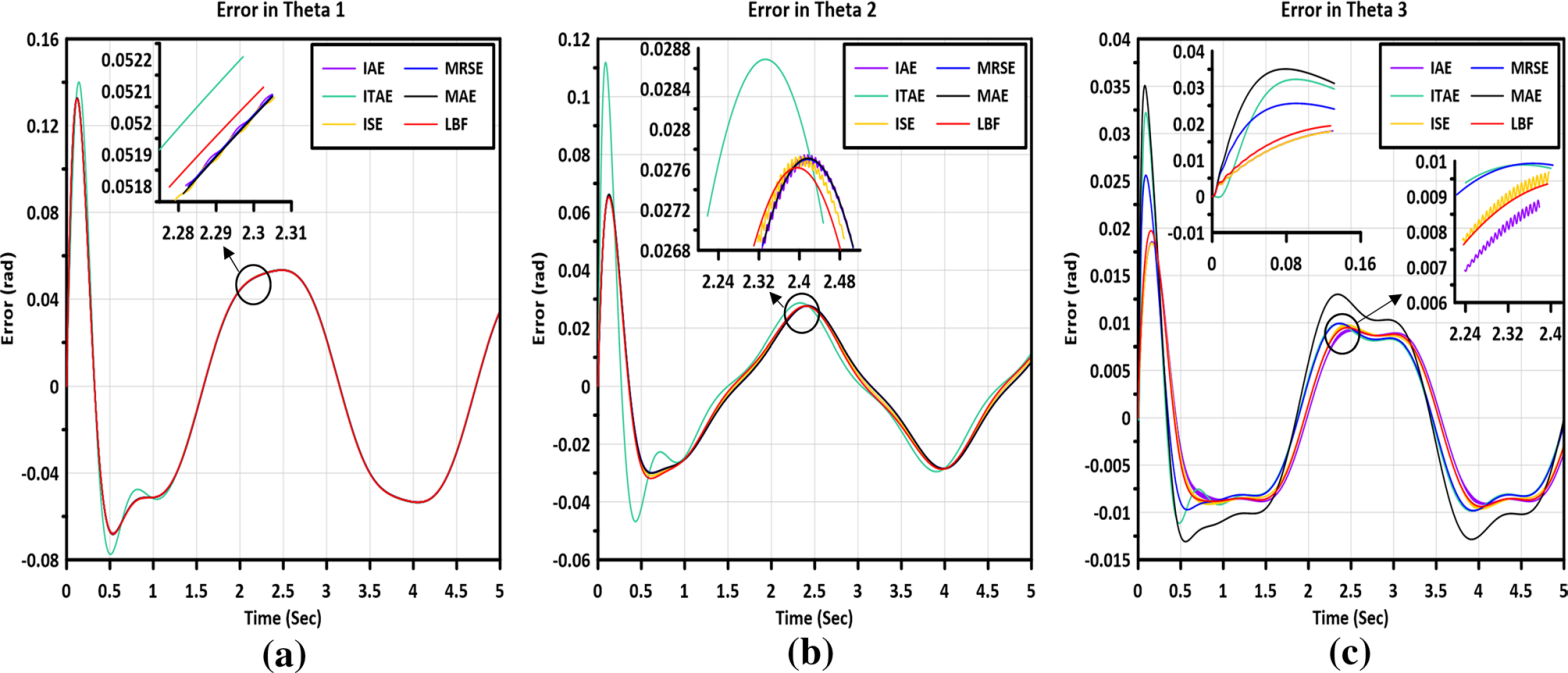
A Comprehensive analysis of all tested objective functions based on the error of measured angles for all links **a**) link (1),** b**) link (2) and **c**) link (3).

Figure 8 illustrates the errors in the trajectories of angles for the three links, using the optimum gains of the controller obtained from the MGABC tuning process. Figure 8a demonstrates that the difference between all tested objective functions is negligible, except for the ITAE, which exhibits a higher peak in the error signals. In Fig. 8b, chattering is observed when the ISE and IAE are employed in the tuning process. However, in Fig. 8c, the new LBF demonstrates superior performance over the other tested functions by eliminating any chattering in the controller, as observed in the ISE and IAE functions. Furthermore, the peaks of the error signals are lower than those observed in ITAE, MRSE and MAE objective functions. The MGABC optimizer yielded the best LBF OF value of $0.10231$ after $40$ iterations. The superiority of the LBF is evident as it demonstrates a significant improvement over various other objective functions in the analysis of tracking trajectory. Specifically, the LBF exhibits a $1.99\%$ improvement over IAE, a $2.22\%$ improvement over ISE, a $48.73\%$ improvement over ITAE, a $4.50\%$ improvement over MAE and a $1.48\%$ improvement over MRSE in terms of the objective function value.

**Figure 9:**
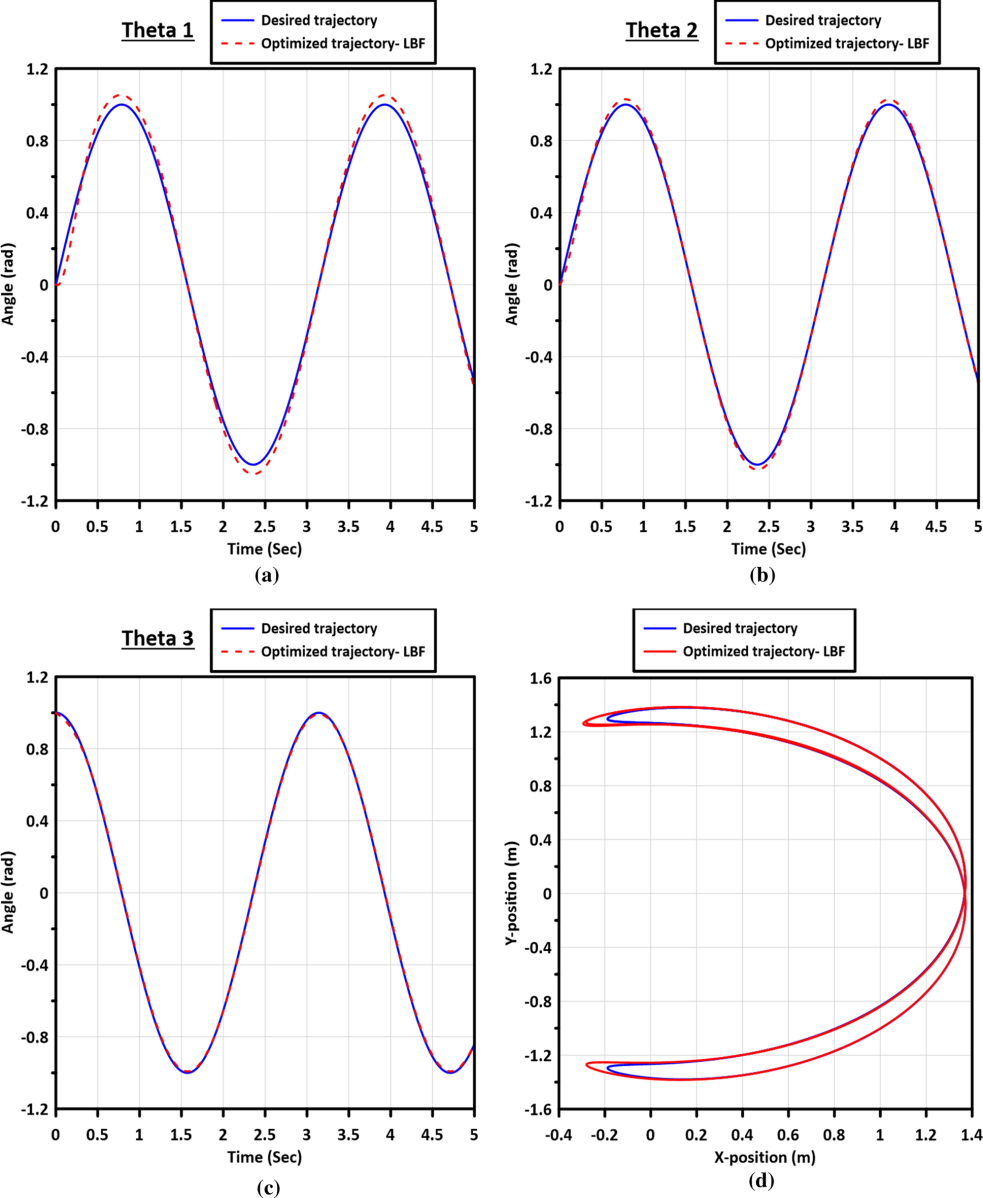
The trajectory tracking curves of the links: (**a**) Link 1, (**b**) Link 2, (**c**) Link 3, and (**d**) the $X-Y$ plot of the end-effector.

Figure 9 presents the trajectory tracking curves of links 1, 2, and 3, along with the $X-Y$ plot of the end-effector. These trajectories are generated using the optimized gains obtained from the Enhanced Artificial Bee Colony with Multi-elite Guidance (MGABC) utilizing the LBF as the objective function during the tuning process.

## Simulation performance evaluation

The complete elimination of uncertainty in the mass of the end-effector and the presence of disturbances, whether measured or unmeasured, is crucial for achieving optimal performance in a control system. These factors can occur independently or simultaneously within the control loop, leading to a degradation in the system's behavior. To address this, the rejection of disturbances and robustness against mass uncertainty have been thoroughly investigated and addressed in this section, aiming to enhance the controller's performance. In addition to the aforementioned investigations, this section will also discuss the impact of joint flexibility on the system. The influence of joint flexibility on the overall performance and behavior of the system will be thoroughly examined and analyzed. The studies conducted for the optimized system using the MGABC algorithm and employing LBF as the objective function are outlined as follows:

### Robustness against disturbance at the controller output

**Figure 10:**
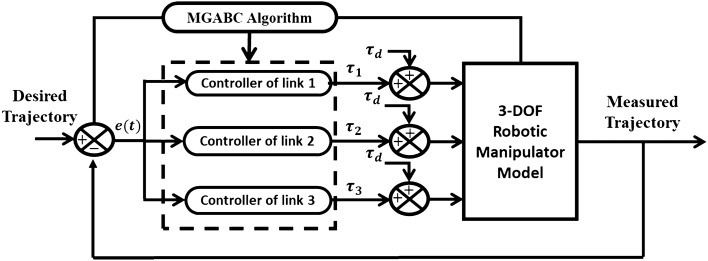
Schematic of robotic system tuning in the presence of disturbance.

A dynamic disturbance signal listed in Eq. (33) is considered and injected at the output of the controller before being applied to the model as shown in Fig. 10.

33 $${\tau }_{d}=A\mathrm{sin}\left(200\pi t\right)+A\mathrm{cos}\left(2t\right)$$

**Figure 11:**
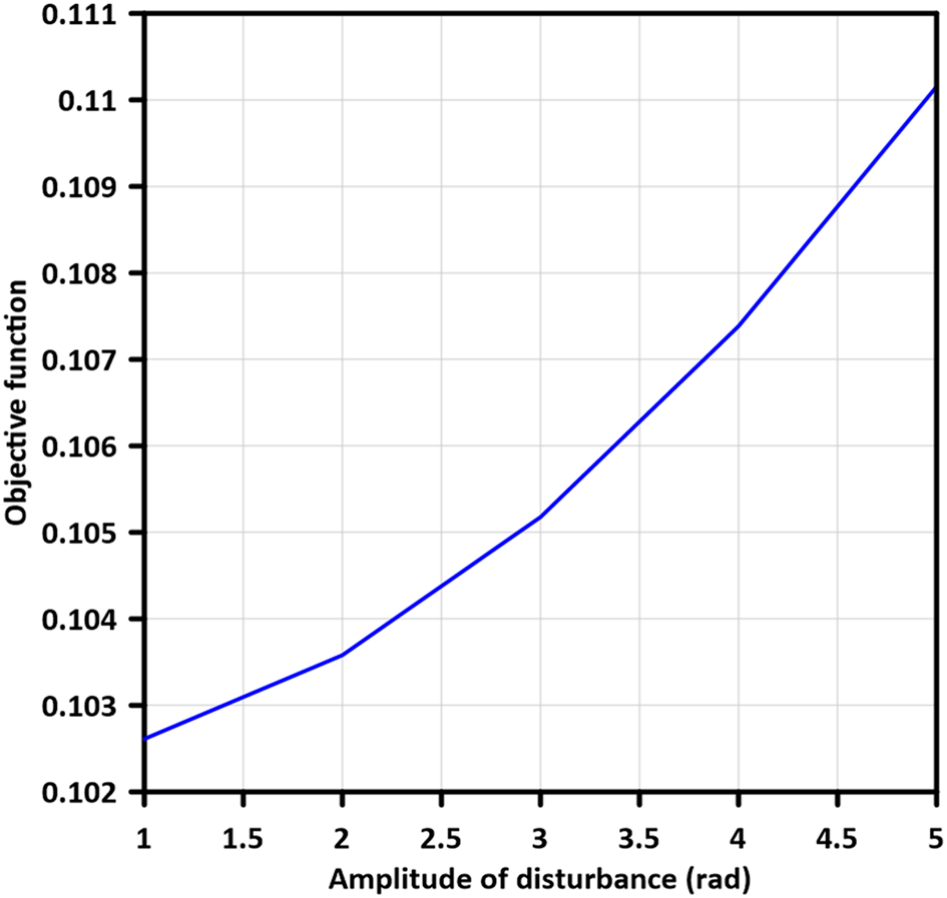
Fluctuations of objective function values on increasing the amplitude of disturbance signal.

The amplitude of the disturbance signal (A) was varied from 1 to 5 with a step of 1 in order to study the elimination of disturbances at the system output. The analysis of the manipulator system's response is presented in Table 4, which provides a comprehensive overview of the obtained objective function (OF) values. Additionally, Fig. 11 illustrates a characteristic curve that represents the fluctuations in OF values corresponding to changes in the amplitude of the disturbance signal.

**Table 4:** Objective function values for variation in amplitude of disturbance signal.

Amplitude	Objective function
	LBF $-$ MGABC
1	0.1026114
2	0.1035804
3	0.1051771
4	0.1073846
5	0.1101554

It becomes evident that the optimized controller exhibits a smooth and stable output which enables the control system to operate effectively for extended durations without compromising performance. The optimized system successfully minimizes the inflation in the objective function and shows its robustness in maintaining OF values within a range of $0.2945\%$ under low disturbance conditions to $7.668\%$ under severe disturbance conditions.

### Robustness against uncertainty in mass of payload

**Figure 12:**
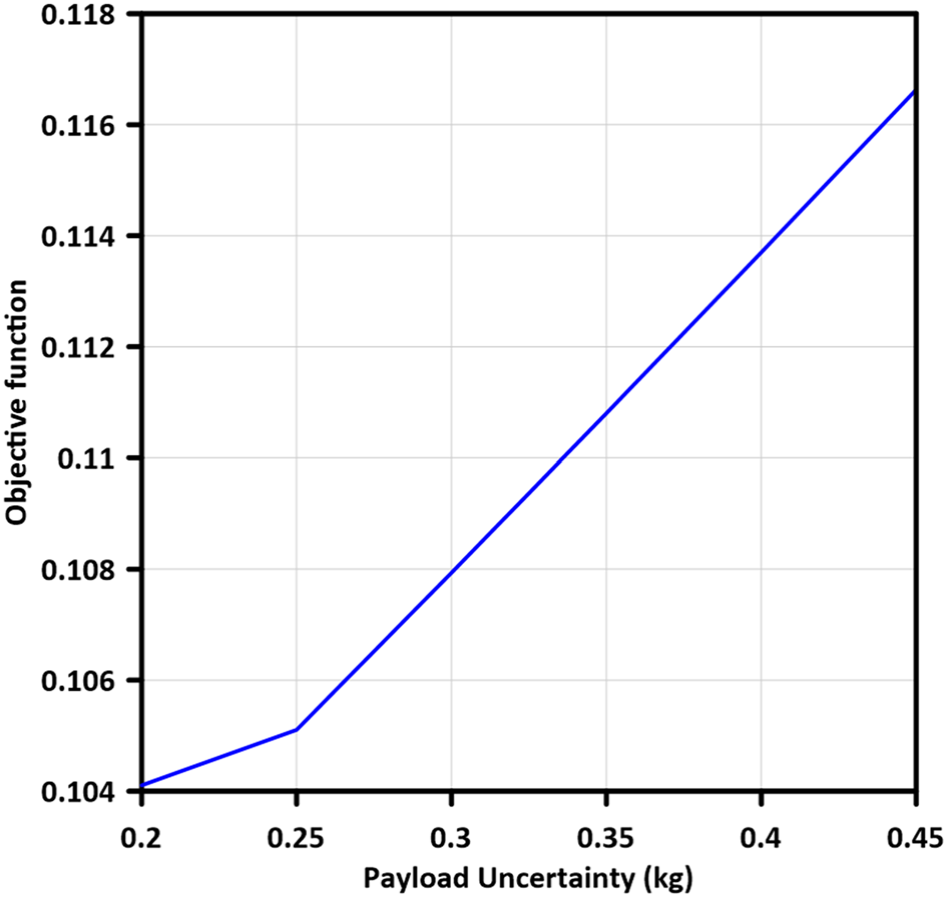
Variation of objective function values with increasing the mass of payload.

A robust controller must have the power to overcome changes made both inside the system and outside it, in most industries, a manipulator's primary duty is to pick up and place objects of varying masses using its end-effector, when the end-effector mass is changed, the controller in real time observes a new system, the effect of end-effector mass variation must be eliminated by a robust controller^4^. The mass of the end-effector was incrementally increased by 0.05 kg, ranging from 0.2 kg to 0.45 kg, in order to evaluate the robustness of the controllers. Table 5 provides a comprehensive list of the obtained objective function (OF) values corresponding to the increasing end-effector mass. Furthermore, Fig. 12 depicts the variations in OF values in relation to the variation in mass of the payload. The optimized system demonstrates effective mitigation of inflation in the objective function, showcasing its robustness in maintaining OF values within a range of 1.755% under low uncertainty conditions to 13.999% under severe uncertainty conditions in the mass of the payload.

**Table 5:** Variation of objective function values with increasing the mass of end-effector.

Uncertainty in mass of payload	Objective function
	LBF $-$ MGABC
0.2	0.1041057
0.25	0.1051057
0.3	0.1079360
0.35	0.1108009
0.4	0.1137002
0.45	0.1166328

### Effect of joint flexibility

When comparing flexible manipulators with their rigid-link counterparts, several notable advantages become evident. Flexible manipulators offer significant benefits, including reduced material usage, lower power requirements, decreased weight, fewer required actuators, and improved maneuverability^45^. Moreover, they enable safer operation in real-world scenarios and higher operating speeds. However, despite these advantages, the widespread application of flexible manipulators in everyday practical settings still presents challenges that must be addressed. The primary objective in controlling a flexible joint is to design a controller that enables a robot link to accurately track a predetermined trajectory or reach a desired position while minimizing link vibrations. Resolving this challenge requires developing control strategies that strike a balance between precise trajectory tracking and minimizing undesired oscillations in the flexible link. This delicate balance is crucial to ensure the effective utilization of flexible manipulators in various real-life applications^2, 46^.

This section aims to assess the effectiveness of the optimized PID controller in mitigating the influence of joint flexibility and achieving the desired trajectory. To thoroughly investigate the impact of flexibility, all three joints of the manipulator are chosen to be flexible. The Simscape model provides valuable resources for this analysis. Specifically, the internal mechanics of each joint are characterized by a spring stiffness of 55 N m/rad and an equivalent viscous damping of 33 N m/rad. These parameters play a crucial role in determining the behavior of the flexible joints and their cumulative effect on the overall system performance.

Table 6 presents a comparison of the OFs values obtained for both rigid and flexible joint configurations, along with the corresponding percentage of inflation. The results demonstrate that the novel LBF outperforms the other error-based functions listed in the literature, exhibiting the lowest inflation. This indicates that the proposed function enables better trajectory tracking and enhanced adaptability in the presence of joint flexibility.

**Table 6:** The percentage of inflation in OFs caused by joints flexibility. Significant values are in bold.

Objective functions	Rigid joints configuration	Flexible joints configuration	Percentage of inflation
IAE	0.3308875	0.6845902	106.895%
ITAE	0.7192567	1.6674362	131.827%
ISE	0.0136527	0.0424705	211.077%
MRSE	0.0354762	0.0927354	161.401%
MAE	0.0429643	0.1483228	245.223%
**LBF**	**0.1023137**	**0.1341760**	**31.141%**

**Figure 13:**
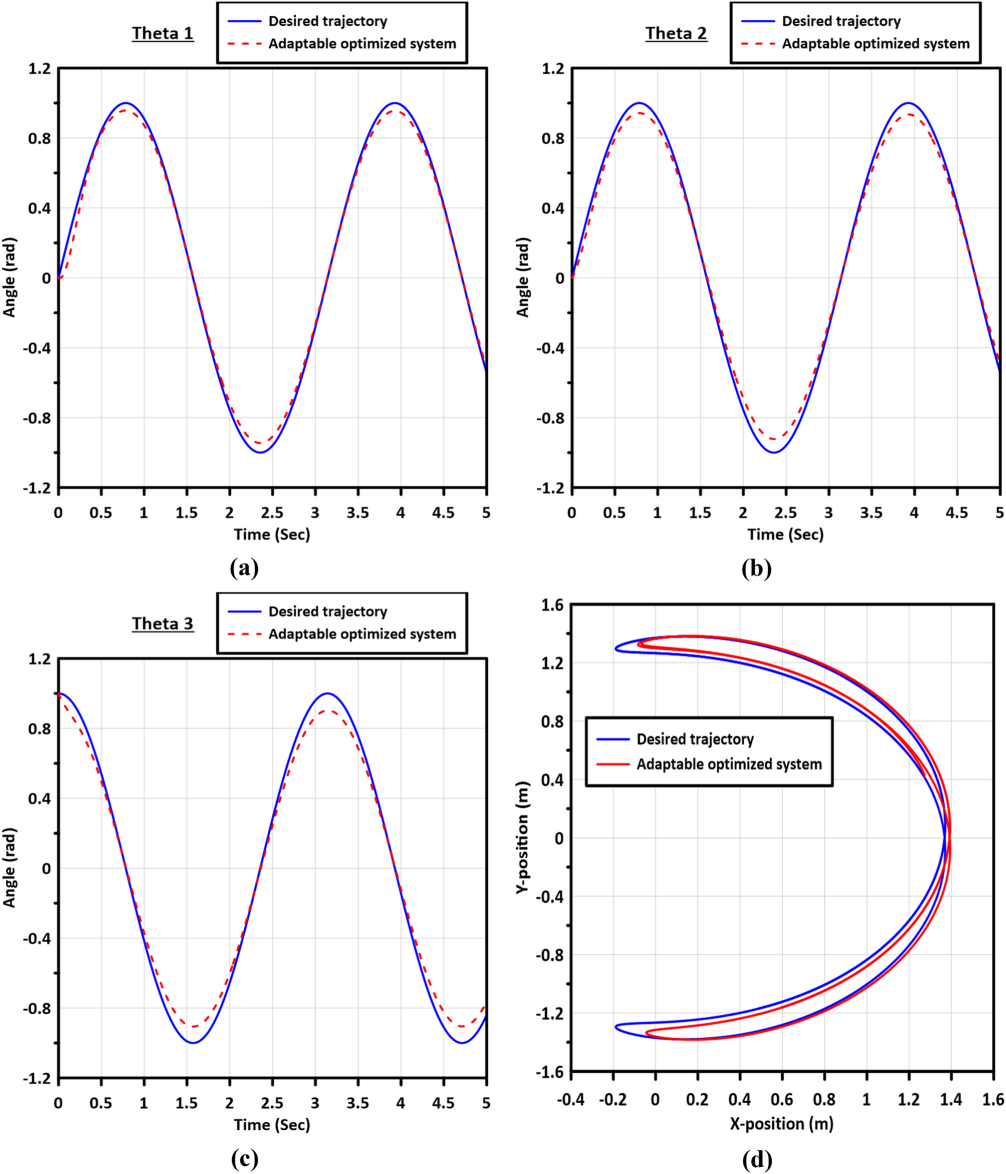
The trajectory tracking curves of the flexible joints of links: (**a**) Link 1, (**b**) Link 2, (**c**) Link 3, and (**d**) the $X-Y$ plot of the end-effector.

**Figure 14:**
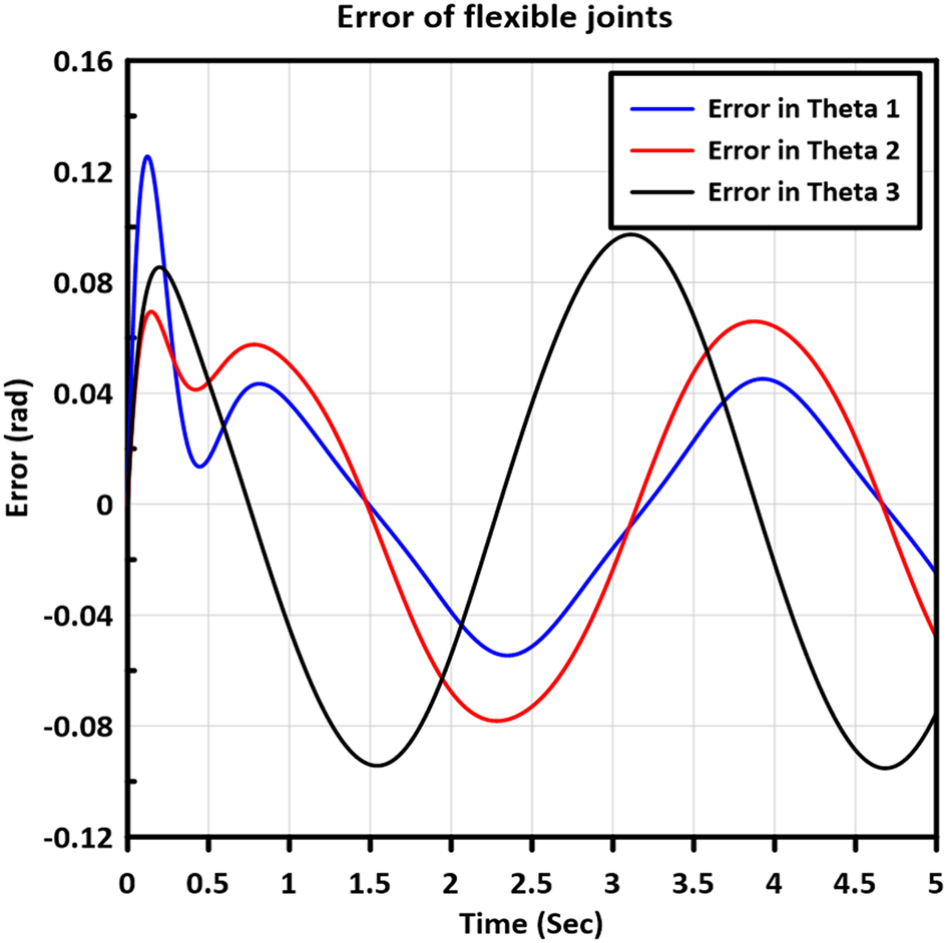
The errors in measured angles of the flexible joints.

Figure 13 displays the trajectory tracking curves of the three flexible joints, as well as the $X-Y$ plot of the end-effector, demonstrating the performance of the controller in adapting to joint flexibility. Additionally, Fig. 14 illustrates the errors in the trajectory angles of the flexible joints, utilizing the optimized gains obtained from the MGABC tuning process. The optimized controller exhibits robustness and adaptability to the impact of joint flexibility, resulting in smooth trajectories with minimal vibration in the movement of the end effector.

## Future work

The present study offers opportunities for further enhancement and extension by exploring additional crucial performance evaluations. The following areas are suggested for future research:

- Investigation of both link and joint flexibility to address potential vibrations in movement, necessitating the development of robust controllers capable of effectively controlling such dynamics.
- Expansion of the analysis to include experimental validation, which will strengthen the credibility and applicability of the study by validating the findings in a real-world setting.
- Conducting a rigorous mathematical analysis to establish the stability of the flexible system, specifically focusing on the selected controller design. This will involve providing formal proofs and theoretical insights into the stability properties of the system.

By addressing these aspects in future work, a more comprehensive understanding of the system's behavior, control strategies, and stability can be achieved.

## Conclusion

In this study, we propose two optimization techniques, namely the basic Artificial Bee Colony (ABC) and the enhanced Artificial Bee Colony with multi-elite guidance (MGABC), for determining the optimal gains of a PID controller in a three-link rigid robotic manipulator system. We introduce a novel objective function, the Lyapunov-based function (LBF), and compare it with established error-based objective functions such as Integral Time Absolute Error (ITAE), Integral Absolute Error (IAE), Integral Square Error (ISE), Mean Root Square Error (MRSE), and Mean Absolute Error (MAE). The purpose of this comparison is to evaluate the effectiveness and performance of the new function in relation to the existing objective functions.

The convergence analysis of all objective functions using the two optimization algorithms reveals that the MGABC outperforms the ABC by effectively exploring the search space and employing diverse exploitation strategies, thus avoiding local optima. The trajectory tracking performance of the optimized controller is examined, and the LBF demonstrates superior performance compared to the other objective functions, with improvements of $1.99\%$ over IAE, $2.22\%$ over ISE, $48.73\%$ over ITAE, $4.50\%$ over MAE, and $1.48\%$ over MRSE in terms of the objective function value. Furthermore, the robustness of the optimized system is evaluated in terms of disturbance rejection and uncertainty in the mass of the payload. The results indicate that the optimized system effectively maintains objective function values within a range of $0.2945\%$ to $7.668\%$ under varying disturbance conditions and $1.755\%$ to $13.999\%$ under different levels of uncertainty in the mass of the payload. Additionally, the adaptability of the optimized controller to joint flexibility is investigated, and it is observed that the controller exhibits robustness and adaptability by generating smooth trajectories with minimal vibration in the movement of the end effector. These findings highlight the effectiveness of the proposed optimization techniques and the LBF in achieving improved trajectory tracking, disturbance rejection, robustness against uncertainty, and adaptability to joint flexibility in robotic manipulator systems.

## Data Availability

The datasets generated during and/or analyzed during the current study are available from the corresponding author on reasonable request.
